# DT389-YP7, a Recombinant Immunotoxin against Glypican-3 That Inhibits Hepatocellular Cancer Cells: An In Vitro Study

**DOI:** 10.3390/toxins13110749

**Published:** 2021-10-22

**Authors:** Hamid Hashemi Yeganeh, Mohammad Heiat, Marek Kieliszek, Seyed Moayed Alavian, Ehsan Rezaie

**Affiliations:** 1 Baqiyatallah Research Center for Gastroenterology and Liver Diseases, Baqiyatallah University of Medical Sciences, Tehran 1435916471, Iran; hamid_hashemi001@yahoo.com (H.H.Y.); mohamad.heiat@gmail.com (M.H.); alavian@thc.ir (S.M.A.); 2 Molecular Biology Research Center, Systems Biology and Poisonings Institute, Baqiyatallah University of Medical Sciences, Tehran 1435916471, Iran; 3 Department of Food Biotechnology and Microbiology, Institute of Food Sciences, Warsaw University of Life Sciences—SGGW, Nowoursynowska 159 C, 02–776 Warsaw, Poland; marek_kieliszek@sggw.edu.pl

**Keywords:** Glypican-3, hepatocellular carcinoma, humanized YP7, diphtheria toxin, new recombinant immunotoxin

## Abstract

Hepatocellular carcinoma (HCC) is one of the high-metastatic types of cancer, and metastasis occurs in one-third of patients with HCC. To maintain the effectiveness of drug compounds on cancer cells and minimize their side effects on normal cells, it is important to use new approaches for overcoming malignancies. Immunotoxins (ITs), an example of such a new approach, are protein-structured compounds consisting of toxic and binding moieties which can specifically bind to cancer cells and efficiently induce cell death. Here, we design and scrutinize a novel immunotoxin against an oncofetal marker on HCC cells. We applied a truncated diphtheria toxin (DT389) without binding domain as a toxin moiety to be fused with a humanized YP7 scFv against a high-expressed Glypican-3 (GPC3) antigen on the surface of HCC cells. Cytotoxic effects of this IT were investigated on HepG2 (GPC3^+^) and SkBr3 (GPC3^−^) cell lines as positive- and negative-expressed GPC3 antigens. The dissociation constant (Kd) was calculated 11.39 nM and 18.02 nM for IT and YP7 scfv, respectively, whereas only IT showed toxic effects on the HepG2 cell line, and decreased cell viability (IC50 = 848.2 ng/mL). Changing morphology (up to 85%), cell cycle arrest at G2 phase (up to 13%), increasing intracellular reactive oxygen species (ROSs) (up to 50%), inducing apoptosis (up to 38% for apoptosis and 23% for necrosis), and an almost complete inhibition of cell movement were other effects of immunotoxin treatment on HepG2 cells, not on SkBr3 cell line. These promising results reveal that this new recombinant immunotoxin can be considered as an option as an HCC inhibitor. However, more extensive studies are needed to accomplish this concept.

## 1. Introduction

Out-of-control growth and proliferation of cells lead to tumor formation [1]. Traditional approaches, such as surgery, radiation therapy, chemotherapy, or a combination of them, in treating malignancies are insufficient and accompanied by many side effects on normal tissues [2,3]. Achieving a new technology or tool for cancer-specific treatment is one of the big topics of conversation in medical research [4,5].

The development of anticancer drugs against specific antigens of cancer cells is one of the new attractive methods for researchers to treat cancers [6,7,8]. As such, in line with this approach, the first, and probably the main, milestone is discovering an appropriated specific antigen as a cancer cell marker [9,10]. GPC3 is a glycophosphatidylinositol (GPI)-anchored cell surface heparan sulfate proteoglycan that is expressed during the early stage of cancer [11], but not in normal adult tissues [12]. GPC3, as an oncofetal antigen, is overexpressed in HCC cells and involved in tumor development through Wnt [13,14], Yap [15], and TGFβ2 [16] signaling pathways. It is detectable on several carcinomas/neoplasms. HCC, yolk sac tumors, thyroid cancer, colorectal carcinoma, gastric carcinoma, pancreatic cancer, and non-small cell lung cancer are the major tissues with GPC3 overexpression. However, evidence has shown that GPC3 is overexpressed in 14% of gastrointestinal tract and pancreatic carcinomas/neoplasms [17]. Meanwhile, HCC with more than 80% expression is taken into account as the most overexpressing tissue [18]. Targeting of such an overexpressed specific antigen on cancer cells by antibodies, or their derivatives, is tempting as an efficient strategy to annihilate cancer cells [19,20,21]. ITs are protein structures consisting of two parts: the toxin and the binding moieties. Herbal or bacterial toxins can be used as a toxin moiety in the structure of ITs. As such, the potent toxins are raised as key agents in cancer treatment, as well as in protection against infectious agents [22]. The binding moiety contains a specific monoclonal antibody or binding fragment, such as the antigen-binding (Fab) or the single-chain fragment variable (scFv) [23,24]. Specific binding of the antibody to the antigen causes the toxin to enter into the cancer cells, subsequently killing the cell by inhibiting protein synthesis. This specific binding diminishes the negative effects of immunotoxins on normal cells [25].

Multiple ITs have been designed against GPC3 of hepatocellular cancer cells [26,27]. One of the problems associated with antibody-based therapies is the secondary immune response to animal-derived therapeutic antibodies as foreign antigens. To overcome this problem, humanized antibodies have been developed in which complementarity-determining regions (CDRs) of the antibody remain intact, and other regions are substituted with their counterparts in human antibodies [28]. Other than humanization, reducing the size of the antibodies can also decrease the secondary immune responses. The functional fragments of antibodies, such as scFvs, nanobodies, and Fab fragments, are much considered in this regard. Yi-Fan Zhang et al. investigated and compered several humanized scFv against GPC3, and provided us with a clear picture of the biological and physicochemical status of existing structures [28]. On the other hand, choosing a meet toxin is another challenge in designing and manufacturing of ITs. The size, function, toxicity, and immunogenicity of the toxin moiety are the bottlenecks for selecting an appropriate toxin.

Summarizing these issues in a valid concept calls for the conduction of a study with the aim of designing and producing an engineered IT structure, and evaluating its bioactivities. Accordingly, after in-silico analysis on different IT constructions composed of previously developed scFvs [28], a truncated diphtheria toxin, and different number of linkers, the best-scored structure was chosen to be produced, and its effect on HCC cells investigated. As such, after the expression and purification of the recombinant IT, its binding affinity and cell toxicity on HepG2 as a hepatocellular carcinoma cell line were investigated.

## 2. Results

### 2.1. Construction Design

**Figure 1:**
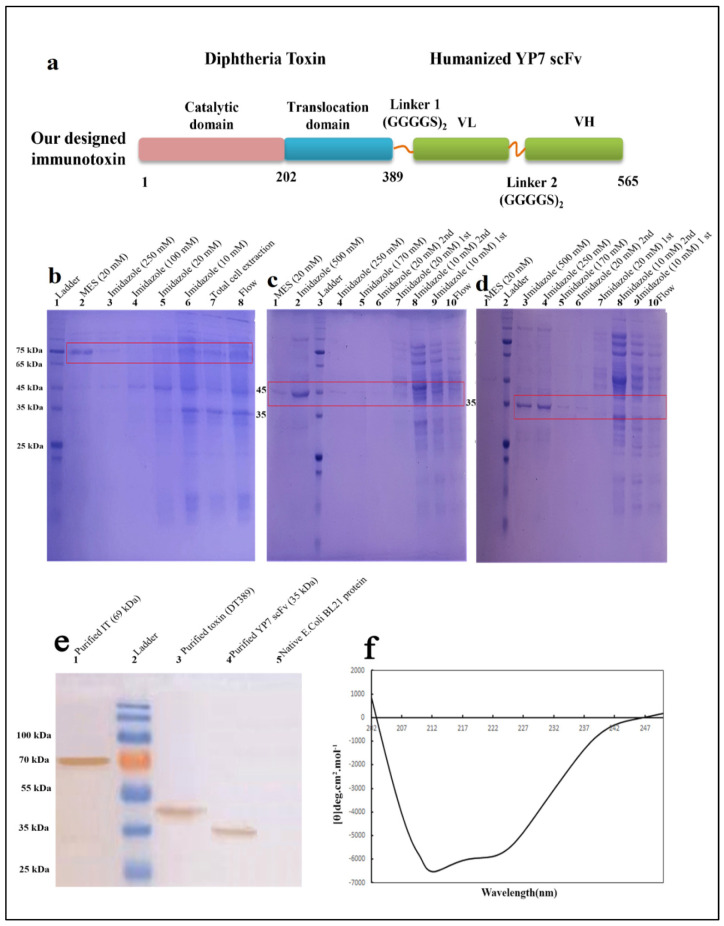
Schematic structures of DT389-(GGGGS)_2_-YP7 immunotoxin. The truncated *Diphtheria* (DT389) was fused to humanized YP7 scFv (developed byY. Zhang et al., 2016) against GPC3 antigen, by two repeats of G_4_S flexible linker (**a**). Purification and validation of proteins using Ni-NTA column and western blotting. Conformational and secondary structure study of IT through CD analysis. Purification of DT389-(GGGGS)2-YP7 IT (**b**), DT389, and humanized YP7 scFv was performed using affinity chromatography and different concentrations of imidazole to achieve the most purified protein of interest on 12% SDS-PAGE. (**b**). 1: ladder, 2: elution buffer containing MES (20 mM), 3: elution buffer containing 250 mM imidazole, 4: elution buffer containing 100 mM imidazole, 5: washing buffer containing 20 mM imidazole, 6: washing buffer containing 10 mM imidazole, 7: total sonicated cell extraction, 8: flow through from column. (**c**). 1: elution buffer containing MES (20 mM), 2: elution buffer containing 500 mM imidazole, 3: ladder, 4: elution buffer containing 250 mM imidazole, 5: elution buffer containing 170 mM imidazole, 6: second washing buffer containing 20 mM imidazole, 7: first washing buffer containing 20 mM imidazole, 8: second washing buffer containing 10 mM imidazole, 9: first washing buffer containing 10 mM imidazole, 10: flow through from column. (**d**). 1: elution buffer containing MES (20 mM), 2: ladder, 3: elution buffer containing 500 mM imidazole, 4: elution buffer containing 250 mM imidazole, 5: elution buffer containing 170 mM imidazole, 6: second washing buffer containing 20 mM imidazole, 7: first washing buffer containing 20 mM imidazole, 8: second washing buffer containing 10 mM imidazole, 9: first washing buffer containing 10 mM imidazole, 10: flow through from column. (**e**). Validating of proteins were performed by western blotting. Results showed that purification of proteins was accurate. 1: purified IT (69 kDa), 2: ladder, 3: purified truncated Diphtheria (DT389) (42 kDa), 4: purified YP7 scFv (35 kDa), 5: total protein extraction of native *E. coli* BL21 without vector. (**f**). Secondary structure of IT using far-CD. Main percentage of secondary structure was dedicated to be α-Helix.

Although in-silico analysis and physic-chemical properties of the three structures were the same, the DT389-(GGGGS)_2_-YP7 IT showed more reliable conformation and structural orientation (Figure 1a). After codon adaptation of DT389-(GGGGS)_2_-YP7 IT based on the *E. coli* strand, the primary expression was optimized and prepared for continued investigation.

### 2.2. Purification and Validation of Proteins

**Figure 2:**
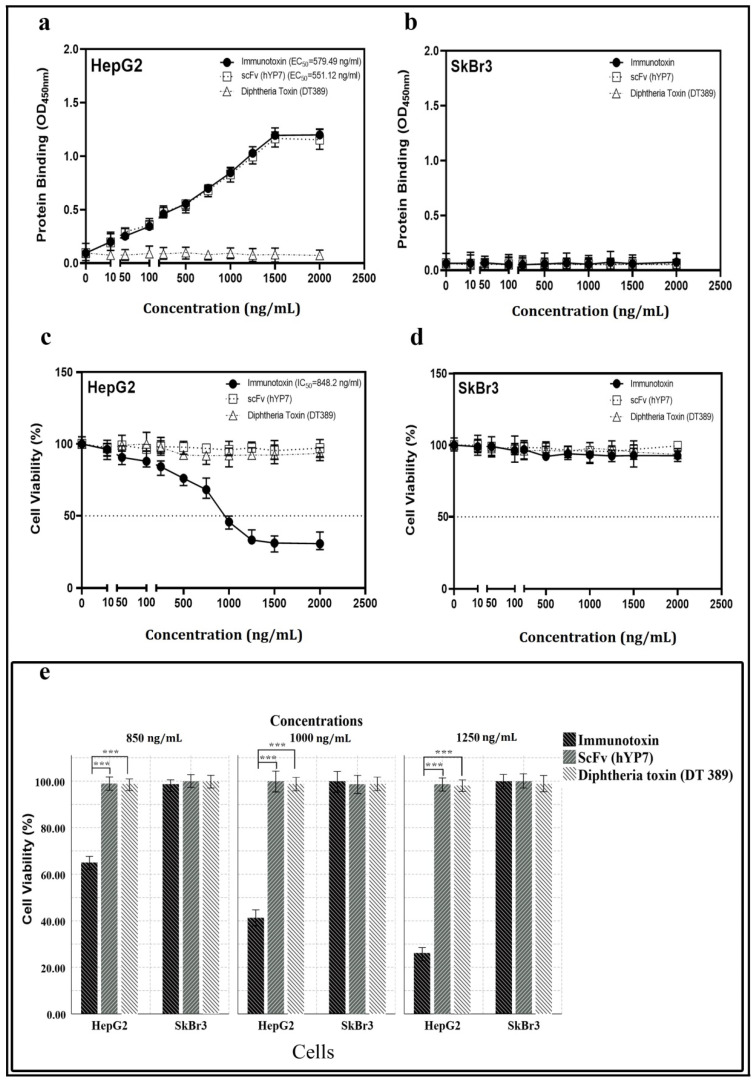
Investigation of binding affinity (**a**,**b**) and toxicity (**e**) of IT, DT389, and YP7 scFv proteins on HepG2 and SkBr3 cell lines after 24 h of treatment. The Kd of immunotoxin, DT389, and YP7 scFv to HepG2 (**a**) and SkBr3 cells (**b**) with different concentrations of each three proteins separately (0, 50, 100, 250, 500, 750, 1000, 1250, 1500, and 2000 ng/mL) using cell-ELISA approach. OD450 nm represented as binding property. Toxic effect of IT, DT389, and YP7 scF on HepG2 cells (**c**) and SkBr3 cells (**d**) with the same concentrations using MTT assay. Decreasing cell viability was observed at higher concentrations of immunotoxin (≥1000 ng/mL) in HepG2 cells. (**e**) Trypan blue assay was used to confirm cell toxicity of immunotoxin. Number of blue-colored cells represented as dead cells in comparison with control group. Results were expressed as the mean ± SD. (*** *p* < 0.001) (*n* = 3).

Purified proteins were analyzed by SDS-PAGE and western blotting (Figure 1a–c). The same purification protocol, as mentioned above, was followed for all three proteins. The most purified protein fraction was eluted in higher concentrations of imidazole (500 mM) and MES (20 mg/mL) buffers. A single band on 12% SDS-Page gel indicated purified proteins. Purification process was carried out separately for DT389-(GGGGS)_2_-YP7 IT (Figure 2b), DT389 (Figure 2c), and humanized YP7 scFv (Figure 2d). To validate purified proteins, the recombinant proteins were detected by Anti His-Tag antibodies and western blot analysis (Figure 2e). Three single bands in 69, 42, and 35 kDa were related to purified IT, truncated *Diphtheria* (DT389), and humanized YP7 scFv, respectively.

The data from conformational analysis indicated that the expression and purification processes have been performed correctly, and SDS-PAGE visualized bands were dedicated to our interested proteins.

Using the GOR IV online server, the secondary structure of IT (DT389-(GGGGS)_2_-YP7) was predicted and compered to experimental data obtained from far-CD (Figure 1f). Experimental analysis on IT structure showed more α-helix and extended strand structures than the in silico predicted structure (Table 1). The percentage of α-helix, extended strand, and random coil in the experimental model were calculated as 40.23%, 29.81%, and 29.96%, respectively. Among these, the percentages of alpha helix and extended strand are slightly more than predicted percentages.

### 2.3. Binding Affinity and Bioactivity

Binding of IT, DT389, and YP7 scFv to HepG2 and SkBr3 cell lines was investigated by cell-ELISA. Results revealed that IT and YP7 scFv bind to HepG2 cells, whereas DT389 could not. The Kd of IT and YP7 scFv was 11.38 nM and 18.02 nM, respectively, on HepG2 (GPC3^+^) cells (Figure 2a). SkBr3 (GPC3) cells as negative control showed no binding attachment for IT, DT389, and YP7 scFv (Figure 2b).

The viability of HepG2 cells was decreased by increasing of IT concentration with an IC50 value of 848.2 ng/mL (Figure 2c), whereas no toxic effect was detected on SkBr3 (Figure 2d). Neither DT389 nor YP7 scFv had a toxic effect on either type of the cancer cell lines. The number of cell death induced by IT, DT389, and YP7 scFv was investigated by trypan blue staining. The results demonstrated that when increasing the concentration of IT, the number of HepG2 dead cells would also be increased (Figure 2e).

### 2.4. Cells’ Morphology

**Figure 3:**
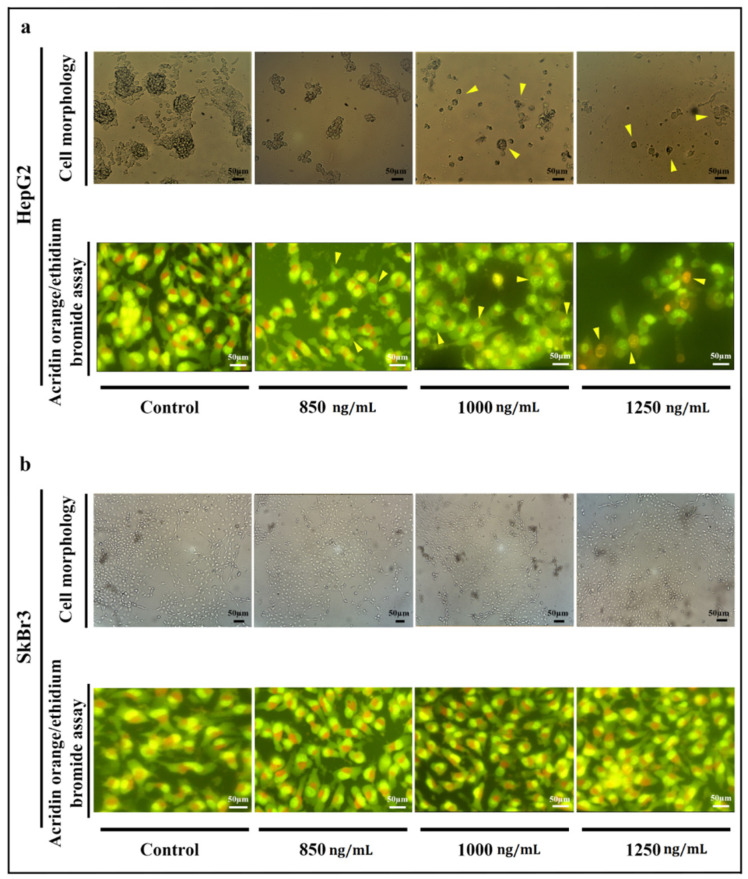
Morphology of HpG2 (**a**) and SkBr3 cell lines (**b**) investigation using optical and fluorescent microscopes. (**a**) Morphology of HepG2 cells was changed after treatment with IT. A decrease in the number of cells and colony-forming ability, cell membrane destruction, and cell shrinkage were observed as main changes (marked cell). Acridine-orange ethidium bromide staining revealed that the rate of apoptosis (early and late) and necrosis were raised up at highest concentrations (1250 ng/mL) of IT compared to control. Cell membrane lobulation and DNA fragmentation were observed in lower concentration of IT (≤1000 ng/mL). (**b**) The same concentrations of IT were applied to SkBr3 cell line, however, no morphologic changes were observed after treatment.

The morphology of HepG2 cells altered after treatment with IT (Figure 3a). Increasing the concentration of IT resulted in a decrease in the number of cells, colony-forming ability, size of cells, and adherent property. In high concentrations (≥1000 ng/mL), cell shrinkage and decreasing cell asymmetry shape were occurred. Acridine orange/ethidium bromide staining of HepG2 cells revealed that IT (850 and 1250 ng/mL) induces apoptosis. Cell membrane lobulation and DNA fragmentation was observed in IT-treated HepG2 cells. All of the SkBr3 cells were intact even in the presence of the highest concentration of IT (Figure 3b). As the results show, DT389 and scFv had no effect on both HepG2 and SkBr3 cell lines.

### 2.5. Apoptosis and Cell Cycle

**Figure 4:**
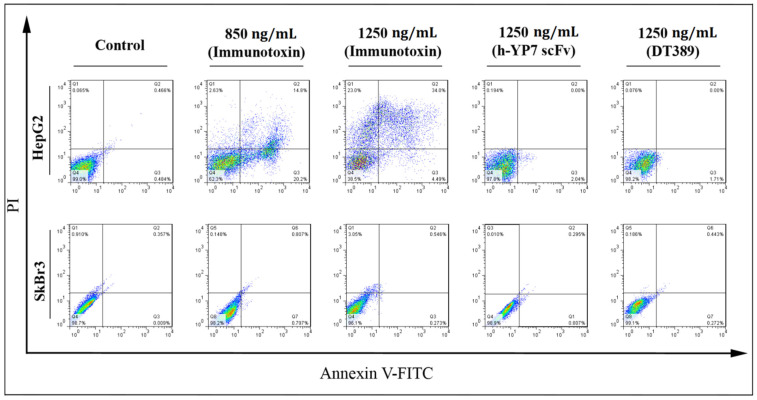
Effect of IT, DT389, and YP7 scFv on apoptosis induction after 24 of treatment using flow cytometry. Categorization of treated cells into necrotic cells (Q1), late apoptosis (Q2), early apoptosis (Q3), and normal cells (Q4). After treatment, HepG2 cells moved into apoptosis and then, necrosis regions. Results were analyzed by FlowJo software. Data have been shown for a single sample.

New recombinant IT activated an apoptosis pathway in HepG2 cells. Twenty-four hours after treatments, HepG2 cells migrated to an apoptosis area division, and the percentage of living normal cell decreased undergoing IT treatment, whereas no decrease of living normal cells in the SkBr3 cell line was observed. The percentage of early and late apoptosis, and necrotic cells, increased compared to control in HepG2 cells when increasing IT concentration (20.2 and 4.49% for early apoptosis, 14.8 and 34.0% for late apoptosis, and 2.63 and 23.0% for necrotic cells were observed after treatment of cells with 850 and 1250 ng/mL of IT, respectively) (Figure 4).

**Figure 5:**
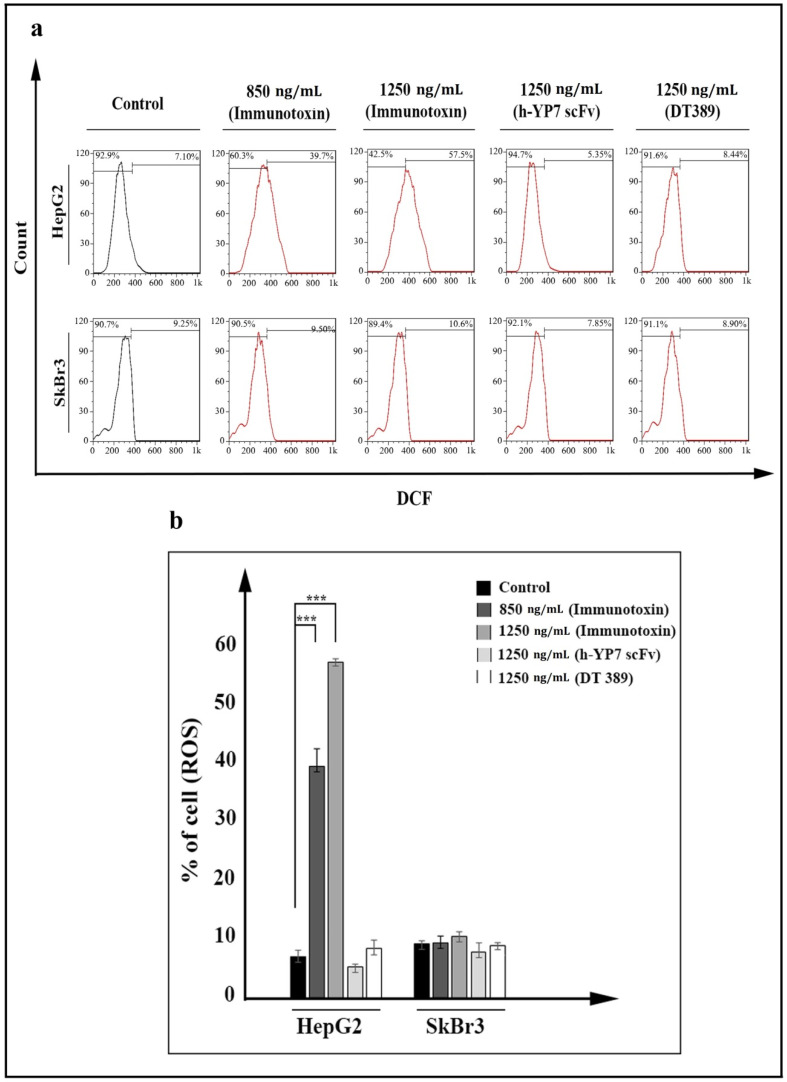
Reactive oxygen species (ROSs) increasing in HepG2 cells treated by IT after 24 h. (**a**) Increasing fluorescent DCF, represented as intracellular ROS in IT treated HepG2 cells (data have been shown for a single sample). (**b**) Percentage of ROS-positive cells was meanly increased at 850 and 1250 ng/mL concentrations of IT in HepG2 cells. Results were analyzed by FlowJo software and expressed as the mean ± SD. (*** *p* < 0.001) (*n* = 3).

Reactive oxygen species (ROSs) in the cells were increased under treatment with IT. ROS participated in the oxidative pathway and consequently, cell death. Increasing dichlorofluorescein (DCF) represented as internal ROS in cells, which is detectable by flow cytometry. By increasing the concentration of IT, the emission intensity in HepG2 cells was increased (Figure 5a), so that 7.1% of pretreatment HepG2 cells with positive internal ROS increased significantly to 39.7% and 57.5% in treated cells with 850 and 1250 ng/mL of IT, respectively (Figure 5b).

**Figure 6:**
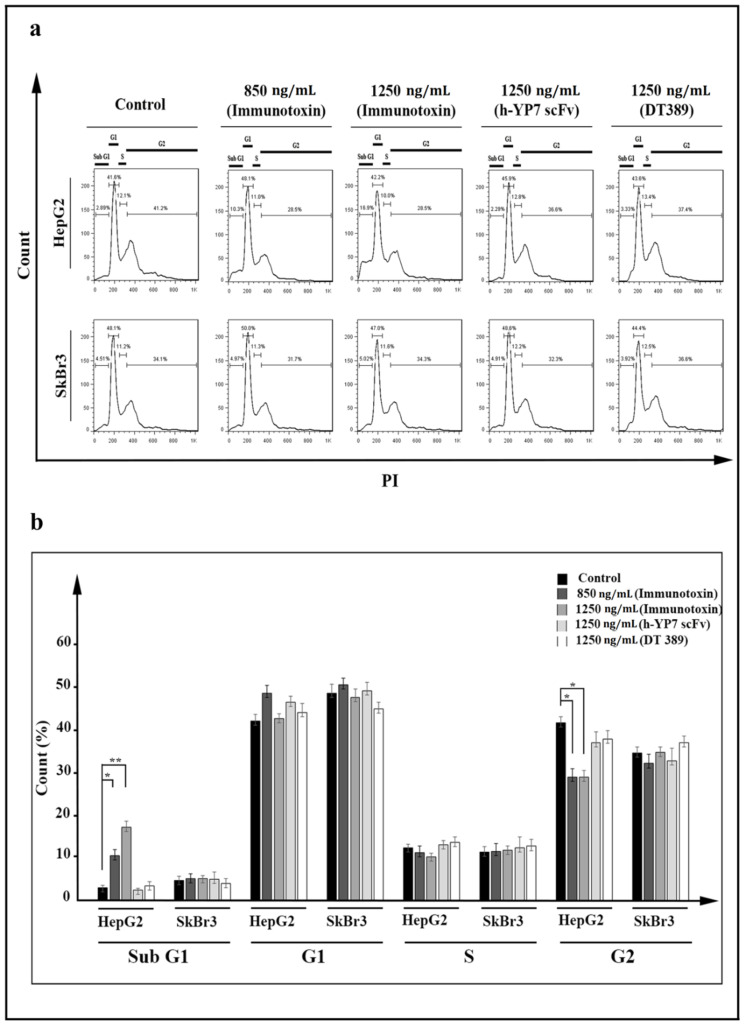
Cell cycle arrest following IT-treatment of HepG2 cells after 24 h. (**a**) Cell cycle was arrested in HepG2 cells treated with IT at G2 phase, but not in SkBr3 cells. Propidium iodide (PI) dye was utilized to stain DNA strands. Different amounts of DNA (single or double strand) were considered to distinguish cells (data have been shown for a single sample). (**b**) Percentage of cells in different phases sub-G1, G1, S, and G2. Distribution of cells in sub-G1 phase increased after IT treatment in HepG2 cells. Results were analyzed by FlowJo software and expressed as the mean ± SD. (* *p* < 0.05 and ** *p* < 0.01) (*n* = 3).

HepG2 cell cycle arrest was also observed under treatment with IT. Based on the intensity of PI staining, cells divided to sub-G1, G1, S, and G2 phases (Figure 6a). The percentage of cells at sub-G1 was increased from 2.89% in the HepG2 control cell line to 10.3% and 16.9% after treatment with 850 and 1250 ng/mL of IT, respectively (Figure 6b). Cells arrest at the G2 phase, and an increase of ROS resulted in more cell apoptosis induction. No cellular effect (neither HepG2 nor SkBr3 cell lines) was observed after treatment with DT389 and YP7 scFv, which indicated that none of them had the capability to inhibit cancer cells on their own.

### 2.6. Cell Movement and Metastasis

**Figure 7:**
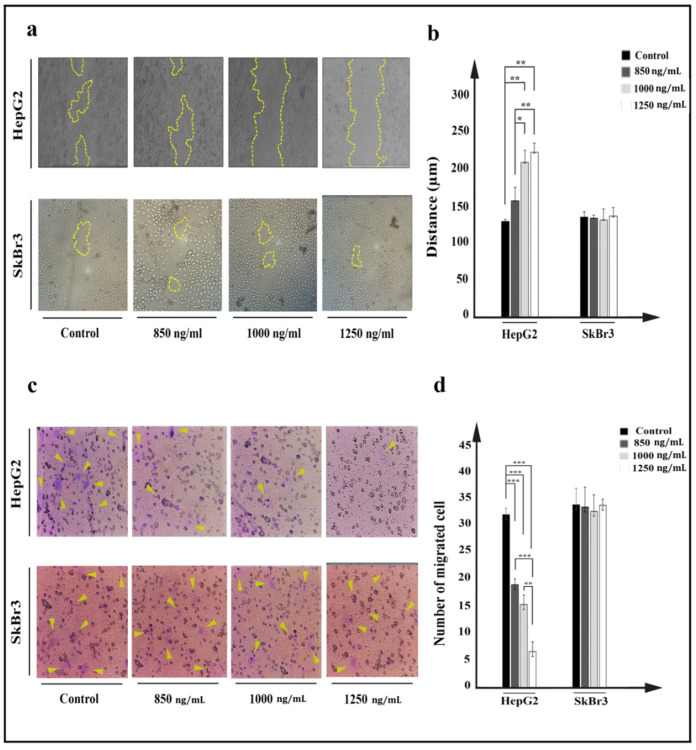
Investigation of effect of IT on HepG2 (**a**,**b**) and SkBr3 cell movement after 24 h treatment with 850, 1000, and 1250 ng/mL of IT. (**a**). Movement of HepG2 and SkBr3 cells was inhibited after treatment. (**b**). Average of distances between edge to edge was measured using ImageJ software, and data were presented as the mean (µm) ± SD. (**c**). After 24 h of migration, passed cells through pores were stained by crystal violet and counted using a fluorescence microscope. (**d**). Passed cells were counted and compared to control in both HepG2 and SkBr3 cell lines. Results were expressed as the mean ± SD. (* *p* < 0.05, ** *p* < 0.01, and *** *p* < 0.001) (*n* = 3).

Movement of HepG2 cells was prevented after IT treatment. Inhibition of cell movement was investigated using scratch and cell invasion assays. Compared to control, higher concentrations of IT (≥1000 ng/mL) caused the related distance of edges to remain intact, and had no inhibitory effect on SkBr3 cells (Figure 7a). The average distance from edges to edge of cell was measured and considered as cell movement. Statistical analysis indicated a significant difference between treated target cells and related controls (Figure 7b).

Cancer cells’ property to change the cytoskeleton and move throughout pores was investigated by a cell invasion assay. A plate with a chamber covered by 8 µm diameter pores was utilized to evaluate property of IT-treated cell to cross through pores, in which passed cells were considered as metastatic cells. The number of passed HepG2 cells was significantly decreased after treatment with IT (Figure 7c). Higher concentrations of IT (≥1000 ng/mL) were more effective in inhibiting cells movement, and in the HepG2 cell line, 32 passed cells in control decreased to 20, 12, and 6 cells at 850, 1000, and 1250 ng/mL concentrations of IT, respectively. No effect was seen on SkBr3 cell line (Figure 7d).

## 3. Discussion

Since 2000, many ITs have been designed to inhibit hepatocellular carcinoma (HCC) cells [29,30]. The variety in scFv and toxin moieties has caused various ITs with different binding affinities and IC50 [14,26,27,31,32].

Pseudomonas exotoxin A (PE), as one of the prominent toxin moieties, is mostly used in IT structures. The intrinsic properties of PE, including, high toxicity, well-known routing and processing, and ease of manipulation, have given it some superiorities over other toxin moieties [33]. PE, possessing the furin cleavable and KDEL-like motifs, facilitates further detaching of the catalytic domain and retrograde transportation to the endoplasmic reticulum (ER), respectively [34].

Diphtheria toxin (DT) is another successful toxin moiety in which the R-domain is substituted with a targeting moiety to construct ITs. DT, like PE, has a furin cleavable moiety, and functionally has shown low IC50. ONTAK, as the only FDA approved IT (certified in 1999), is a DT-based IT against cutaneous T-cell lymphoma.

The other arm of IT is a targeting moiety. To choose the best targeting moiety, it is essential to find a fit target. Evidence has revealed that CPC3 would be an ideal antigen to be used as an IT target in HCCcells. Hitherto, multiple ITs (e.g., HN3, HS20, and YP7) have been introduced against GPC3 [12,16,17,18]. GPC3 is related to the Yap and Wnt signaling pathways. Findings have demonstrated that HN3 and HS20 have inhibitory effects on both pathways. However, YP7 (a mouse mAb which identified the C-terminal of GPC3) has shown no effect on neither Wnt nor Yap signaling pathways. Nevertheless, all of them have shown anti-tumor activity in vivo [14].

In the present study, several IT structures were designed based on in silico analysis. Among all, the DT389-(GGGGS)_2_-YP7 immunotoxin was chosen as the main structure, and its structural stability was studied in our laboratory. Considered IT and its components (DT389 and YP7 scFv) were expressed and purified separately using immobilized metal affinity chromatography (IMAC). Western blot and far-CD analyses were performed to confirm accuracy of purified protein and the secondary structure. The Kd for IT and YP7 scFv was 11.39 nM and 18.02 nM, respectively, and IC50 for IT on HepG2 cells was 848.2 ng/mL. No binding affinity for DT389 was observed neither for HepG2 nor SkBr3 cell lines. Besides, DT389 and YP7 scFv showed no cytotoxic effect on HepG2 and SkBr3 cell lines. A lethal effect of IT on the HepG2 cell line was significantly manifested, and at higher concentrations of IT (≥1000 ng/mL), the morphology and colony forming ability of cells were disrupted (Figure 3a).

Previously, the cytotoxic effect of YP7-based immunotoxins has been approved by Zhang and their colleagues, and they designed humanized anti-GPC3 scFvs, namely hYP7 and hYP9.1 fused with PE, and investigated their affinity and cytotoxicity. They grafted the combined KABAT/IMGT complementarity determining regions (CDRs) of mouse scFvs into a human IgG germline framework. In spite of a very similar scFv sequence of mouse YP7-, YP8-, YP9-, and YP9.1-PE38 immunotoxins, the two YP7-PE38 and YP9.1-PE38 showed higher efficiency and performance and subsequently, were chosen to be humanized. In humanized immunotoxins, the binding affinity of hYP9.1-PE38 was better, but hYP7-PE38 was more cytotoxic [28].

DNA fragmentation, as the major feature of early apoptosis cells [35], was observed in HepG2 cells treated with IT. At 1250 ng/mL concentration of IT, the HepG2 cell was promoted to activate the necrosis pathway, as well as the apoptosis pathway. Flow cytometry data analysis also indicated the apoptosis activation and necrosis development in HepG2 cells, but not in SkBr3 cell line.

Protein synthesis inhibition following inactivation of elongation factor 2 (EF2) by the immunotoxin is associated with loss of mitochondrial membrane potential (MtMP), and promotes cells death. Furthermore, it has been revealed that IT induces apoptotic proteins such as Bax and DNA fragmentation factors, cytochrome c (Cyt c) release, and ultimately, caspase-dependent cell death [36,37,38].

As shown, reactive oxygen species (ROSs) were increased in IT-treated HepG2 cells. It has been demonstrated that ROSs were increased in cancer cells treated with IT through inhibition of antioxidant pathways, such as the KEAP1-NRF2 pathway [39]. In the following, ROSs with their extremely reactive and toxic properties, can oxidize cellular components, and have synergetic effects on cell destruction and death. [40,41].

Data of cell cycle analysis obtained from flow cytometry revealed that our recombinant IT arrests the HepG2 cell cycle at the G2 phase. Previous studies have been shown that ITs can impress cell cycles at G1/S phases [42,43,44]. Correlation among DNA fragmentation, apoptosis, and cell cycle arrest has been extensively investigated. Increasing Bax as an apoptotic factor represented a transcriptional factor for p53, which is considered a tumor suppressor by inducing cell cycle arrest and cell apoptosis [30,45,46]. Generally, in addition to direct effect of IT on the activation cell apoptosis, increasing intracellular ROS and arresting cells into the G2 phase will confer it the maximum toxic effects.

Metastasis of primary cancer cells to other organs makes cancer cells difficult to cure, so inhibition of cell movement would be a promising way to overcome the challenges of metastatic cancers [47]. Results of a scratch assay and cell invasion assay revealed that the treatment of HepG2 cells with IT reduces the cell movement and invasion (Figure 5). Reducing proteins that participate in cell movement and metastasis is considered a consequence of protein synthesis inhibition followed by IT treatment [48]. As is mentioned above, YP7 is not able to block signaling pathways through binding to GPC3 [14]. As such, it could be reasonable that the reduced movement can only be attributed to cell death. However, more investigations are needed to reach a perfect concept in this regard.

## 4. Conclusions

In conclusion, DT389-(GGGGS)_2_-YP7 recombinant IT demonstrated a toxic effect on the HepG2 cell line. Cell morphology alteration, colony-formation ability impairing, ROS levels elevation, induction of apoptosis and cell cycle arrest and cell movement, and metastasis inhibition were the major effects of IT treatment. DT389-(GGGGS)_2_-YP7 immunotoxin could be considered as a novel recombinant protein to inhibit HCC cells with high expressions of GPC3. However, this underling scientific effort calls for further studies to respond to all questions around this recombinant protein.

## 5. Materials and Methods

### 5.1. Construction Design

A variety of structures of ITs were constructed, composed of a truncated diphtheria toxin (residues 1-389; DT389) (Expasy accession no. P00588) [49] and different Yi-Fan Zhang’s humanized anti-GPC3 scFvs (YP7, YP8, YP9 and YP9.1) [28], using credible in-silico software. This preliminary phase of studies focused on investigating the effectiveness of different G_4_S linkers between toxin and scFv moieties on secondary and three-dimensional structures, stability, flexibility, solubility, and physic-chemical properties. In the end, the structure with the highest score (DT389-(GGGGS)_2_-YP7) was selected as best IT candidate. The nucleotide sequence of the selected construct was synthesized in PET 21a+ and transformed to the *Escherichia coli* strain for recombinant expression. Apart from IT, the toxin and YP7 scFv moieties were separately produced for further analysis. Two- and three-dimensional structure analysis, as well as the degree of stability in all recombinant proteins was investigated.

### 5.2. Production, Purification, and Validation of Recombinant Protein by Affinity Chromatography and Western Blotting

IT (DT389-(GGGGS)_2_-YP7), truncated diphtheria (DT389), and scFv (humanized YP7) were produced (under the induction condition of 1 mM isopropyl β-D-1-thiogalactopyranoside (IPTG) at 37 °C for 6 h incubation) and purified using a nickel-nitrilotriacetic acid (Ni–NTA) affinity column. Buffers containing 10, 20, 100, 170, 250, and 500 mM of imidazole and MES (2-(*N*-morpholino) ethane sulfonic acid) buffer were utilized for column calibration, washing, and elution. All eluted fractions were collected separately and analyzed by 12% sodium dodecyl sulfate polyacrylamide gel electrophoresis (SDS-PAGE). The concentrations of recombinant proteins were obtained using a Bradford assay. The purified recombinant proteins were validated by western blotting, and the three proteins were applied to the separate wells of SDS-PAGE. Then, proteins were transferred to a nitrocellulose membrane and blocked with tris-buffered saline (TBS) containing 0.05% Tween 20 (TBST) and 5% skim milk for 2 h in a shaker (SPEED rpm) at room temperature. After removing the blocking buffer and washing with PBST, the nitrocellulose membrane was incubated overnight at 4 °C with 1:2000 diluted horseradish peroxidase (7)-conjugated Anti His-Tag antibodies (Sigma, Berlin, Germany). The membrane was washed three times with TBST, and DAB (3,3′-Diaminobenzidine) substrate was added to detect the proteins of interest.

### 5.3. Circular Dichroism Analysis

The secondary structures (alpha-helix, beta-sheet, beta-turn, or some other conformations (e.g., random coil)) of recombinant proteins were determined by a ultraviolet circular dichroism (CD) spectrum. To do this, the purified proteins (0.3 mg/mL) in buffer were used, and analyzed by a CD spectrum at 180 to 240 nm.

### 5.4. Cell Lines and Culture

Human HepG2 (GPC3^+^) hepatocellular carcinoma (HCC) and human SkBr3 (GPC3^−^) breast cancer cell lines were obtained from the cell bank department of Pasture institute (Tehran, Iran), and cultured in supplemented Roswell Park Memorial Institute (RPMI-1640) medium (Gibco, Carlsbad, CA, USA) at 37 °C in 5% CO_2_ incubation condition. The media had been supplemented with 10% fetal bovine serum (FBS) (Gibco, CA, USA), and 1% antibiotic (50 U/mL of penicillin and 50 µg/mL streptomycin).

### 5.5. Cytotoxic Effect of Immunotoxin

An MTT assay was performed to investigate the cytotoxic effect of the immunotoxin. HepG2 and SkBr3 cell lines were seeded into the wells of a 96-well plate at a density of 15,000 cells in 200 µL culture medium. After overnight incubation at 37 °C in 5% CO_2_, cells were separately exposed with different concentrations (10, 50, 100, 250, 500, 750, 1000, 1250, 1500, 2000 ng/mL) of DT389-(GGGGS)_2_-YP7 immunotoxin, DT389, and humanized scFv (YP7). Then, cells were incubated at 37 °C for 24 h. Thereafter, 30 µL of 3-(4,5-dimethylthiazol-2-yl)-2,5-diphenyltetrazolium bromide (MTT) stock solution (5 mg/mL) was added into each well, and the incubation was continued for 4 h. Afterward, dimethyl sulfoxide (DMSO) was added to dissolved formed formazan crystals by living cells. Subsequently, the absorbance of the solution was measured at 570 nm by an ELISA microplate reader (Spectra MAX Plus; Molecular Devices, San Jose, CA, USA).

A trypan blue assay was also used to determine the immunotoxin-mediated cell death. After 24 h of cell treatments with 850, 1000, and 1250 ng/mL of each immunotoxin, DT389, or YP7 scFv proteins, cells were harvested and mixed with a trypan blue stain [1;1], and counted using a hemocytometer and inverted microscope (Nikon Instruments Inc., Tokyo, Japan). Blue cells were considered as death cells.

### 5.6. Cell Morphology Analysis

To investigate the morphology of cells, 120,000 cells/well were seeded in a 12-well plate and after treatment with different concentrations of immunotoxin (850, 1000, and 1250 ng/mL), the cell morphology was analyzed using an inverted microscope. Acridine orange/ethidium bromide staining of treated cells was carried out to detect apoptotic and necrotic cells. An amount of 250 µL/well of acridin orange and ethidium bromide (24 mg/mL for both of them) was added to cells and incubated for 3 min. Cells were washed twice with PBS (1×) and analyzed using fluorescence microscopy.

### 5.7. Cell Enzyme-Linked Immunosorbent Assay (Cell-ELISA)

A cell-ELISA test was used to investigate the affinity binding of recombinant proteins to GPC3^+^ cells. In this regard, 15,000 cells/wells were seeded into microtiter plates (Nunc-ImmunoPlates^®^ Maxisorp, Frankfurt, Germany) and incubated at 37 °C upon reaching 70% confluence. After cell fixation by formaldehyde (10% *v*/*v*) and blocking with PBS-BSA solution (6% *w*/*v*), the cells were treated with different concentrations of DT389-(GGGGS)_2_-YP7 immunotoxin, DT389, and humanized YP7 scFv, as mentioned in the MTT assay. After washing with PBST four times (PBS (1×) containing 0.05% Tween-20), the diluted Anti-His Tag antibodies (1:16,000 in PBST) were added into each well. The plates were then incubated for 60 min at 37 °C. Thereafter, each well was washed four times with PBST to remove non-specific antibodies. Finally, 3,3′,5,5′-tetramethylbenzidine (TMB-H_2_O_2_; Sigma-Aldrich, St. Louis, MO, USA) solution was added, and color development proceeded for 20 min before the addition of a stop solution (2 M H_2_SO_4_). The absorbance value of each well was measured at 450 nm using a microplate reader (Bio-Rad, Hercules, CA, USA). The resulting data were expressed in terms of OD values.

### 5.8. Annexin V/PI Apoptosis Detection

Apoptotic effects of recombinant proteins were investigated on HepG2 and ShBr3 cells using an Annexin V/propidium iodide (PI) assay. Cells were seeded into a 12-well plate incubated at 37 °C to reach 60% related confluence. Cells were treated with different concentrations of immunotoxin (850 and 1250 ng/mL), DT389 (1250 ng/mL), and YP7 scFv (1250 ng/mL), and incubated at 37 °C for 24 h. Cells were harvested and washed twice with PBS (1×), and resuspended in binding buffer. Categorization of cells into four horizons, including alive, early and late apoptotic, and necrotic cells, was determined by an Annexin V/PI staining kite. Fluorescein isothiocyanate (FITC)–conjugated Annexin V/PI solution was added and incubated at room temperature for 30 min. Apoptosis analysis was carried out using FACSCalibur™ Flow Cytometer (BD Biosciences, San Jose, CA, USA).

### 5.9. Cell Cycle Investigation

Analysis of cell distribution in different phases of cell cycle (subG1, G1, S, and G2) was performed using PI staining and a flow cytometric assay. After treatment of cells with concentrations of immunotoxin (850 and 1250 ng/mL), DT389 (1250 ng/mL), and YP7 scFv (1250 ng/mL) defined for 24 h, cells were harvested and fixed with ethanol 70% (*v*/*v*) for 30 min. Then, the cells were washed twice with PBS (1×) and propidium iodide (10 µg/mL) was added to determine cell distribution using a FACSCalibur™ Flow Cytometer. Results were analyzed by FlowJo™ Software version 7.6.1.

### 5.10. Quantification of ROS in Cells

Dichloro-dihydro-fluorescein diacetate (DCFH-DA) passes through cell membrane, and is oxidated to convert to fluorescent molecule dichlorofluorescein (DCF) by reactive oxygen species (ROSs). To perform this test, the same cell counts were seeded and treated as cell cycle analysis. Treated cells were incubated with 10 µM of DCFH-DA for 1 h. The emission fluorescent was measured using a flow cytometer, and its data were analyzed by FlowJo software version 7.6.1.

### 5.11. Cell Migration

Migration of cells was investigated using scratch and cell invasion assays. For the scratch assay, a 12-well plate was coated by cells overnight and then treated with 850, 1000, and 1250 ng/mL of immunotoxin. A central cell-free line was created using a sterile yellow tip, and cells were permitted to migrate for the next 24 h. Distance between scratch edges was calculated using ImageJ 1.48 software.

The cell invasion assay was performed using a Transwell™ plate with a special chamber containing 8 µm diameter pores at the bottom. After treatment of cells with 850, 1000, and 1250 ng/mL concentration of immunotoxin for 24 h, 10,000 treated cells of each concentration were added into the upper chamber containing 100 µL cell culture medium with 2% FBS. In the lower part, cell culture medium with 10% FBS was added, and cells were permitted to move through pores for 48 h. Passed cells were fixed using ethanol 70% (*v*/*v*), and stained with 0.1% (*w*/*v*) crystal violet for 5 min. Stained cells were investigated using an inverted microscope. The process of cell migration was explored by inverted microscope visualization.

### 5.12. Statistical Analyses

Data were presented as means ± standard deviations. All experiments were repeated in triplicate. Using IBM SPSS statistics 26, all data were analyzed. Significant statistical differences among the groups, obtained from fit statistical tests, are presented with * *p* < 0.05, ** *p* < 0.01, and *** *p* < 0.001 in histograms.

## Figures and Tables

**Table 1:** Result of secondary structure obtained from fat CD.

Type of Fusion Protein	Alpha Helix	Extended Strand	Random Coil
DT289-(GGGGS)2-YP7	40.23	29.81	29.96

The analyzed data are displayed in percentage.

## Data Availability

Not applicable.
